# Molecular Analysis of Cerebrospinal Fluid Tumor-Derived DNA to Aid in the Diagnosis and Targeted Treatment of Breast Cancer Brain Metastasis

**DOI:** 10.3390/diseases13100336

**Published:** 2025-10-11

**Authors:** Michael Youssef, Alexandra Larson, Vindhya Udhane, Viriya Keo, Kala F. Schilter, Qian Nie, Honey V. Reddi

**Affiliations:** 1 Department of Neurology, Department of Hematology and Oncology, UT Southwestern Medical Center, Dallas, TX 75235, USA; michael.youssef@utsouthwestern.edu; 2 Belay Diagnostics, Chicago, IL 60607, USA; alarson@belaydiagnostics.com (A.L.); vudhane@belaydiagnostics.com (V.U.); vkeo@belaydiagnostics.com (V.K.); kschilter@belaydiagnostics.com (K.F.S.); snie@belaydiagnostics.com (Q.N.)

**Keywords:** breast cancer, biomarkers, personalized therapy, liquid biopsy, cerebrospinal fluid, brain metastasis

## Abstract

A woman in her 40s with a history of ER/PR+, HER2-negative breast cancer presented with a seizure three years after mastectomy. Magnetic resonance imaging (MRI) revealed a right caudate head mass, which was concerning for either high-grade glioma or metastatic disease, but biopsy was deemed too high risk. Cerebrospinal fluid (CSF) tumor-derived DNA (tDNA) analysis by next-generation sequencing (NGS) was ordered, revealing a gain-of-function variant in *PIK3CA*, *ERBB2* copy number gain, and high aneuploidy, findings consistent with breast cancer brain metastasis. Based on these results, the patient was treated with stereotactic radiosurgery (SRS) followed by trastuzumab deruxtecan, a HER2-targeted therapy. This case highlights the diagnostic and therapeutic value of CSF tDNA analysis in central nervous system (CNS) lesions when biopsy is not feasible. The report also illustrates how clonal evolution, such as acquired *ERBB2* amplification, can occur in metastatic disease and influence treatment decisions.

## 1. Introduction

Primary central nervous system (CNS) tumors can begin either in the brain or spinal cord and encompass >100 distinct types including glioma and medulloblastoma [1] or they can metastasize from other tissue to the CNS with metastasis from lung, breast, and skin being the most common [2]. Although the true incidence of brain metastases has been challenging to determine, it is estimated that approximately 40% of patients with solid tumors will develop CNS metastatic disease [3]. Metastasis to the brain occurs in an estimated 20–40% of breast cancer patients with a median overall survival of 1 month when untreated and 1–2 years with treatment [4]; it is the second most common cause of brain metastasis. The risk of brain metastasis in individuals with stage IV breast cancer is usually highest for those with more aggressive types of breast cancer such as HER2-positive or triple negative breast cancer than luminal subtypes [5]. Between 5% and 10% of breast cancer patients with CNS metastases include two distinct entities: cerebral parenchymal involvement (brain metastases, BM) and infiltration of the cerebrospinal fluid-filled leptomeningeal space (leptomeningeal disease, LMD) [3]. As a result, CNS metastasis represents a unique evolutionary trajectory compared to extracranial metastases. CNS involvement remains a major clinical challenge, partly due to the blood–brain barrier (BBB), with the diagnosis of intracranial brain metastases relying heavily on magnetic resonance imaging (MRI), particularly in cases when lesion location is unamenable to biopsy [6]. Complicating diagnosis, there is considerable overlap of radiologic findings for intracranial metastatic lesions and glial neoplasms [6]. Tumor molecular profiling can offer significant insight into pathogenesis and tissue of origin, particularly in CNS neoplasms [7,8]. Since tumor-derived DNA (tDNA) from CNS disease is shed and circulated in cerebrospinal fluid (CSF), next-generation sequencing (NGS) of CSF tDNA has gained recognition as an impactful liquid biopsy tool [9,10], particularly in the context of negative CSF cytology which is the gold standard, however with limited sensitivity [11].

Belay Diagnostics launched its Summit™ [12] and Vantage™ [13] liquid biopsy tests to potentially increase the efficacy of diagnosis of CNS malignancies and decrease the uncertainty and morbidity associated with the current standard of care, which involves neurosurgical procedures. Summit demonstrated an analytical sensitivity of 96% for single-/multi-nucleotide variants (SNVs/MNVs) and insertions/deletions (indels) at a 95% limit of detection of 0.30% variant allele frequency (VAF). Analytical sensitivity for chromosomal arm level aneuploidy was 83% at abs(log2r) of 0.09 limit of detection [12]. Clinical sensitivity across a cohort of 113 CNS tumors was demonstrated to be 88% with a specificity of 95% [12]. The Belay Vantage™ assay evaluates *MGMT* promoter methylation status in CSF of individuals with known or suspected central nervous system tumors. The assay uses quantitative polymerase chain reaction (qPCR) post enzymatic conversion of tDNA extracted from CSF and has an analytical sensitivity of 95.5% and specificity of 100% [13]. Since launch, both tests have demonstrated significant clinical utility in informing the diagnosis and management of primary and secondary CNS malignancies, as documented in the literature, obviating biopsy [14] and informing diagnosis of metastatic CNS disease [15].

Since CNS metastasis represents a unique evolutionary trajectory, accurate diagnosis to inform treatment and management is key to disease progression and overall survival. The identification of metastasis driver mutations in CSF tDNA guides treatment, expanding treatment options and enabling targeted therapies as applicable. In breast cancer brain metastasis (BCBM), surgical resection or stereotactic radiosurgery (SRS) potentially followed by whole brain radiation therapy (WBRT) are utilized for local management [4]. Systemic therapy use, however, can be dependent on molecular characterization, such as HER2 expression status and mutational status of *ERBB2*, *PIK3CA*, *AKT1*, *PTEN*, and *ESR1* [4,16], particularly in the primary tumor. Uncovering the molecular landscape of BCBM using CSF can be pivotal for the selection of targeted therapies, especially in cases when tumor tissue is not accessible, obviating biopsy and preventing unnecessary morbidity. Herein we present a case of woman in her 40s with an intracranial lesion deemed too high-risk for biopsy. CSF tDNA analysis with the Belay Summit and Vantage tests was used alongside MRI to aid in BCBM diagnosis and therapeutic decision making, highlighting the role of CSF liquid biopsy to inform diagnosis and treatment of CNS tumors, particularly BCBM.

## 2. Materials and Methods

The Belay Diagnostics Summit [12] and Vantage [13] tests were used for CSF tDNA analysis. These CSF based liquid biopsy assays are CLIA/CAP approved for the evaluation of simultaneous detection of both gene level variants, single and multi-nucleotide variants, including insertions and deletions (SNVs, MNVs and indels) in 32 genes and chromosome-arm-level alterations (aneuploidy) [12] along with *MGMT* promoter methylation [13]. Summit [12] uses both duplex NGS to detect genomic gene-level variants and low-pass whole-genome sequencing (WGS) to assess aneuploidy in a single CSF sample with low input tDNA. Vantage [13] interrogates 12 CpG sites in the *MGMT* promoter using enzymatic conversion followed by qPCR.

Both tests can be performed either independently or simultaneously using a single tDNA input. Post sequencing, variants are evaluated to identify those that are clinically significant in that they are actionable (have associated targeted therapies/clinical trials or diagnostic/prognostic relevance) in line with recommendations from the Association for Molecular Pathology (AMP)/American Society of Clinical Oncology (ASCO)/College of American Pathologists (CAP) guidelines for the interpretation of somatic variants [17]. For this particular case, tumor-derived DNA extracted from 12 mL of CSF submitted to Belay for testing via Summit and Vantage was used to evaluate for clinically significant variants to determine next steps in treatment and management of disease.

## 3. Case Presentation (Results)

**Figure 1:**
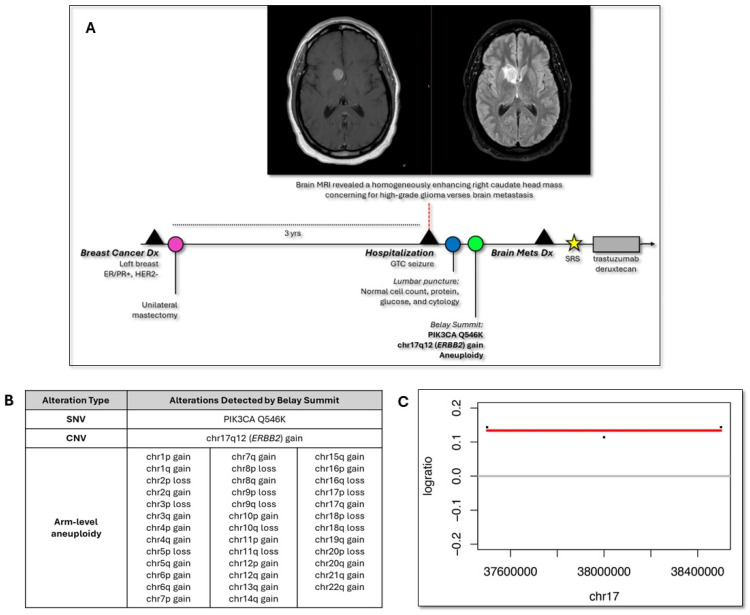
Summary of clinical events and cerebrospinal fluid (CSF) liquid biopsy findings. (**A**) Graphical chronology of the case including diagnoses and key clinical events (black triangles), surgery (pink), lumbar puncture (blue), Belay Summit CSF tumor-derived DNA (tDNA) analysis (green), stereotactic radiosurgery (SRS) (yellow), and systemic therapy (gray box) as well as brain magnetic resonance imaging (MRI) (post-contrast T1-weighted image displayed on the left, T2 FLAIR on the right). Diagnosis, Dx; Generalized tonic–clonic, GTC; Metastatic, Mets. (**B**) Single nucleotide variants (SNV), copy number variants (CNV), and chromosomal arm-level aneuploidy detected in CSF tDNA by Belay Summit. (**C**) Using low pass whole genome sequencing of tDNA, copy number gain of ERBB2 was detected in CSF at a log2 ratio of 0.145 as shown in the ichorDNA plot.

A woman with a known history of ER/PR+, HER2-negative carcinoma of the left breast status post unilateral mastectomy in her 40s (Figure 1A) showed no signs of disease recurrence for about three years. No family history of cancer was noted, and her personal medical history was otherwise significant for polycystic ovarian syndrome and a history of seizures beginning in her 40s with no established cause. She presented to the emergency department for a generalized tonic–clonic (GTC) seizure about three years after her first epileptic episode (and cancer diagnosis). Per standard of care, brain MRI was ordered which showed a homogeneously enhancing right caudate head mass with intralesional hemorrhage, increased perfusion, and mild surrounding vasogenic edema. Additional CT evaluation of the chest, abdomen, and pelvis was negative. The location of the lesion was determined to be too high-risk for biopsy, and the patient was hospitalized with a referral for a neuro-oncology consult two days later.

During the consultation, the physical exam and neurological assessment were normal. Upon review of imaging, the brain lesion was found to be concerning for high-grade glioma versus BCBM. To address the differential diagnosis, the patient underwent a lumbar puncture (LP) which yielded normal cell count, protein, and glucose levels as well as negative CSF cytology, with no malignant cells being detected. About 12 mL of CSF was sent to Belay Diagnostics for genomic profiling via Summit [12] and Vantage [13] tests to inform diagnosis in the context of positive neuroimaging and negative CSF cytology results. Summit reported a variant of clinical significance in the PIK3CA gene, Q546K at a variant allelic frequency of 0.1% as well as a high level of chromosomal arm level alterations including gain of chr17q12 (containing ERBB2), with Vantage being negative for *MGMT* promoter methylation (Figure 1B,C).

While the lesion identified on brain imaging was more concerning for high-grade glioma rather than metastatic disease, the CSF liquid biopsy findings more closely aligned with breast cancer metastasis. PIK3CA Q546K is a well-characterized oncogenic variant that results in constitutive activation of the PI3K/AKT/mTOR cell proliferation signaling pathway [18]. Oncogenic variants in *PIK3CA* are one of the most common genetic alterations in breast cancer with an estimated frequency of 26% [19]. While *PIK3CA* alterations can be seen in high-grade glioma, they are a less common biomarker in that primary CNS tumor type [20]. Gain of *ERBB2* is also one of the most recurrent copy number gains in breast cancer brain metastases and is associated with HER2 overexpression [21,22,23]. Lastly, high aneuploidy is indicative of chromosomal instability, a key driver of metastasis across cancer types [24].

Given the CSF tDNA findings and the patient’s cancer history, it was determined that the brain lesion observed on neuroimaging was most likely due to metastatic breast carcinoma and not a high-grade glioma as previously suspected. The patient was started on trastuzumab deruxtecan (T-DXd), a HER2 antibody-chemotherapy conjugate, with Gamma Knife radiation to the mass. MRI post-radiation showed overall favorable response, patient is currently stable on T-DXd with a proposed MRI in a month to evaluate for treatment efficacy and response.

## 4. Discussion

Diagnosis of CNS tumors, particularly brain metastasis, relies significantly on neuroimaging and CSF cytology in the context of clinical information. The limited sensitivity observed with both methods can be improved by including molecular profiling of CSF tDNA to inform diagnosis of CNS tumors [9] as observed in this case presentation. Recent studies have shown the impact of liquid biopsy to inform diagnosis of CNS tumors, including the Belay Summit test, which achieved a clinical sensitivity of 100% in metastatic breast cancer (*n* = 5) during assay validation [12]. A cohort study evaluating six cases of BCBM demonstrated 100% sensitivity for detecting oncogenic genomic alterations in CSF circulating tumor DNA (ctDNA). This study also critically included paired genomic profiling of primary tumor tissue, plasma, and other metastatic sites, revealing key differences in the genomic variants identified in each specimen type [25]. This finding illustrates how clonal evolution promotes disease progression and potentially highlights opportunities for targeted treatment, especially against CNS-localized driver mutations. Other studies report 55–100% sensitivity when investigating the use of CSF ctDNA analysis to detect brain metastasis from varying primary tumor types [9,26]. While careful pattern analysis of MRI findings and clinical course are essential for characterizing CNS neoplasms, the use of CSF tDNA analysis in this patient helped clarify the differential diagnosis, distinguishing between primary and metastatic disease.

Beyond confirming tumor of origin in this case, CSF ctDNA analysis revealed potential genomic therapeutic targets. Notably, the patient’s original pathology of the primary tumor was found to be HER2-negative, though a copy number gain of *ERBB2* was detected in CSF tDNA several years later. Recent investigations have demonstrated successful prediction of HER2 status per the ASCO/CAP guidelines with high specificity using NGS to detect copy number alterations of *ERBB2* [27]. ASCO/CAP recommendations for HER2 testing in breast cancer were last updated in 2023 and reaffirmed use of immunohistochemistry for best practice [28]. *ERBB2* analysis by NGS nevertheless shows great promise as a clinical tool that can be performed on liquid biopsy with a relatively quick turnaround time.

HER2 amplification has been detected in CSF tumor cells of patients with LMD spreading from various solid tumors including breast, upper GI, and lung [29]. Switches in HER2 status (HER2 flip) between primary breast tumors and brain metastasis or disease recurrence have been reported in the recent literature, highlighting that CNS metastatic disease can evolve and have different biological features compared to the primary tumor. In a matched case study of 136 BCBM patients, 54% of HER2-negative primary breast cancer cases were found to have detectable HER2 expression in brain metastatic site resections [22]. Using *ERBB2* copy number analysis by NGS, another study of 20 BCBM patients with matched primary tumor and brain resections reported that *ERBB2* alterations were acquired in 20% of HER2-negative cases [23]. Notably, HER2-positive status has been identified as a predictor of neurologic death in patients with BM (*n* = 1218, study completed prior to FDA approval of T-DXd) [30].

Alongside research that demonstrates the prevalence and potentially poor prognosis of HER2-positive brain metastasis, recent efforts have further developed HER2-targeted therapies for BCBM [31]. Identifying HER2 amplification in the CSF can provide opportunities for targeted anti-HER2 therapies such as intrathecal trastuzumab. Additionally, serial monitoring of HER2 status in CSF can show changes over time, influencing treatment decisions. Following detection of *ERBB2* gain in the present case, T-DXd was administered following SRS as a preferred systemic therapy for HER2-positive BCBM per NCCN guidelines [31]. In clinical trials, T-DXd exhibited up to a 71% intracranial objective response rate (iORR) in HER2-positive BCBM [32,33]. A similar iORR of 73% has been observed in newly diagnosed BCBM patients treated with a combination therapy of pyrotinib, tyrosine kinase inhibitor, and capecitabine [34]. With the great promise of HER2-targeted therapies, it becomes more critical to identify HER2 positivity in BCBM, a dynamic molecular feature that can arise with CNS involvement.

The most recent 2021 World Health Organization (WHO) classification of tumors of the CNS edition 5 advocates for the adoption of integrated diagnostic approaches containing both classical histology and tissue-based tests along with molecular characterization [35]. Additionally, the recent update to the National Comprehensive Cancer Network (NCCN) guidelines, version 3.2024 states that molecular analysis using next generation sequencing is the preferred approach for the pathologic evaluation of CNS tumors, as it screens for multiple diagnostic and prognostic mutations in one test [31]. In line with previously demonstrated clinical utility to obviate biopsy [14] and inform diagnosis of metastatic disease [15], this case report further highlights the clinical utility of the Belay Summit and Vantage tests in increasing the sensitivity of CSF liquid biopsy by providing actionable genomic information for BCBM. The addition of genomic testing as recommended by WHO and NCCN does indeed increase the sensitivity of CSF liquid biopsy testing and provides a path forward for the patient.

## 5. Conclusions

Contributing to the growing body of literature on liquid biopsy clinical utility, this case report exemplifies the use of CSF tDNA analysis to clarify differential diagnosis, confirming secondary CNS disease and further characterizes malignancy using genomic information to steer treatment. The finding of molecular results using CSF liquid biopsy in the context of negative CSF cytology to inform diagnosis and treatment solidifies the clinical utility of the Belay Summit test. Identification of a HER2 status switch in this patient’s brain metastasis supports clonal evolution of metastatic disease and allowed for timely delivery of targeted therapy. In the era of precision medicine, effective detection of spatial and temporal molecular landscapes is crucial for tackling an ever-evolving disease. CSF liquid biopsy offers clinicians a minimally invasive way to gain insight into BCBM and determine the best course of treatment for their patient.

## Data Availability

The original contributions presented in this study are included in the article. Further inquiries can be directed to the corresponding author.
